# Complete chloroplast genome sequence of *Hedychium coronarium*

**DOI:** 10.1080/23802359.2019.1659114

**Published:** 2019-09-06

**Authors:** Dong-Mei Li, Chao-Yi Zhao, Gen-Fa Zhu, Ye-Chun Xu

**Affiliations:** Guangdong Key Lab of Ornamental Plant Germplasm Innovation and Utilization, Environmental Horticulture Research Institute, Guangdong Academy of Agricultural Sciences, Guangzhou, China

**Keywords:** *Hedychium coronarium*, Zingiberaceae, *Hedychium*, chloroplast genome, phylogenetic analysis

## Abstract

The first complete chloroplast genome of *Hedychium coronarium* (Zingiberaceae) was reported in this study. The *H. coronarium* chloroplast genome was 163,949 bp in length and comprised a pair of inverted repeat (IR) regions of 29,780 bp each, a large single-copy (LSC) region of 88,581 bp and a small single-copy (SSC) region of 15,808 bp. It encoded 141 genes, including 87 protein-coding genes (79 PCG species), 46 tRNA genes (28 tRNA species), and eight rRNA genes (four rRNA species). The nucleotide composition was asymmetric (31.68% A, 18.35% C, 17.74% G, 32.23% T) with an overall AT content of 63.92%. Phylogenetic analysis showed that *H. coronarium* was classified into a monophyletic group within the genus *Hedychium* in family Zingiberaceae.

*Hedychium coronarium* is one species of the genus *Hedychium* (Zingiberaceae), which distributes predominantly in southeast, south to southwest of China, south to southeast Asia and Australia (Wu et al. 2016). *Hedychium coronarium* pseudostems are 1–3 m tall; leaves sessile; ligule 2–3 cm, membranous; leaf blade oblong-lanceolate, 20–40 × 4.5–8 cm, adaxially glabrous, abaxially finely pubescent or thinly hairy, base acute, apex long acuminate (Wu and Larsen 2000). *Hedychium coronarium* plants are cultivated for their ornamental, medicinal and aromatic values (Wu and Larsen 2000; Wu et al. 2016). Within genus *Hedychium*, no complete chloroplast genome for *Hedychium* species has been reported so far (Li et al. 2019a), hindering species identification of *Hedychium* species based on chloroplast genomes.

*Hedychium coronarium* was collected from Guangzhou, Guangdong province, and stored at the resource garden of environmental horticulture research institute (specimen accession no. Hc2015), Guangdong academy of agricultural sciences, Guangzhou, China. Total chloroplast DNA was extracted from about 100 g of fresh leaves of *H. coronarium* using the sucrose gradient centrifugation method (Li et al. 2012). Chloroplast DNA (accession no. HcDNA2017) was stored at −80 °C in Guangdong Key Lab of Ornamental Plant Germplasm Innovation and Utilization, Environmental Horticulture Research Institute, Guangdong Academy of Agricultural Sciences, Guangzhou, China. Library construction was using Illumina (Illumina, CA, USA) and PacBio (Novogene, Beijing, China) sequencing, respectively. The Illumina and PacBio sequencing data were deposited in the NCBI sequence read archive under accession numbers SRR8147014 and SRR8143983, respectively. After trimming, 69.4 M clean data of 150 bp paired-end reads and 0.65 M clean data of 8–10 kb subreads were generated. The chloroplast genome of *H. coronarium* was assembled and annotated using the reported methods (Li et al. 2019b). The annotated complete chloroplast genome sequence of *H. coronarium* was submitted to the GenBank (accession no. MK262736).

The complete chloroplast genome of *H. coronarium* was 163,949 bp in length and comprised a pair of inverted repeat (IR) regions of 29,780 bp each, a large single-copy (LSC) region of 88,581 bp, and a small single-copy (SSC) region of 15,808 bp. It was predicted to contain a total of 141 genes, including 87 protein-coding genes (79 PCG species), 46 tRNA (28 tRNA species), and eight rRNA (four rRNA species). Twenty gene species occurred in double copies, including eight PCG species (*ndhB*, *rpl2*, *rpl23*, *rps7*, *rps12*, *rps19*, *ycf1*, and *ycf2*), eight tRNA species (*trnA-UGC*, *trnH-GUG*, *trnI-CAU*, *trnI-GAU*, *trnL-CAA*, *trnV-GAC*, *trnR-ACG*, and *trnN-GUU*), and all four rRNA species (*rrn4.5*, *rrn5*, *rrn16*, and *rrn23*). All these 20 gene species were located in the IR regions. The *ycf1* gene crossed the boundary of SSC-IRb region, whereas the *rps12* gene was located its first exon in the LSC region and other two exons in the IRs regions. In addition, 10 PCG genes (*atpF*, *ndhA*, *ndhB*, *rpoC1*, *petB*, *petD*, *rpl2*, *rpl16*, *rps12*, and *rps16*) and six tRNA genes (*trnK-UUU*, *trnG-GCC*, *trnL-UAA*, *trnV-UAC*, *trnI-GAU*, and *trnA-UGC*) had a single intron, whereas two other genes (*ycf3* and *clpP*) possessed two introns. The nucleotide composition was asymmetric (31.68% A, 18.35% C, 17.74% G, and 32.23% T) with an overall AT content of 63.92%. The AT contents of the LSC, SSC, and IR regions were 66.15%, 70.48%, and 58.85%, respectively.

To obtain its phylogenetic position within family Zingiberaceae, a phylogenetic tree was constructed using single nucleotide polymorphisms (SNPs) arrays from available 16 species chloroplast genomes using *Costus viridis, Costus pulverulentus*, and *Canna indica* as outgroup taxa. The SNP arrays were obtained as previously described method (Li et al. 2019a). For each chloroplast genome, all SNPs were connected in the same order to obtain a sequence in FASTA format. Multiple FASTA format sequences alignments were carried out using ClustalX version 1.81 (Thompson et al. 1997). A maximum-likelihood phylogenetic tree (Figure 1) was constructed using the SNPs from 16 chloroplast genomes alignment result with MEGA7 (Kumar et al. 2016). As shown in the phylogenetic tree (Figure 1), *H. coronarium* was classified into a monophyletic group (bootstrap value = 97%) within the genus *Hedychium* in family Zingiberaceae with available SNPs.

**Figure 1.**
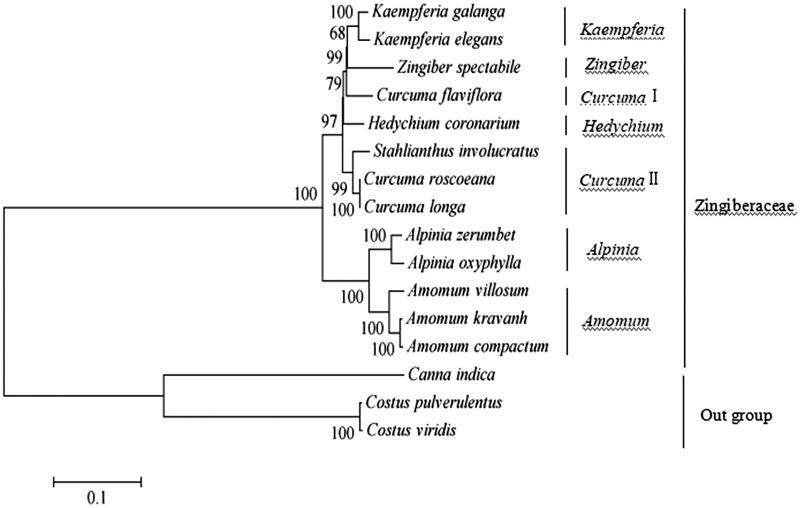
Phylogenetic tree constructed with single nucleotide polymorphisms arrays from 16 species chloroplast genomes using maximum-likelihood method. The bootstrap values were based on 1000 replicates and are indicated next to the branches. Accession numbers: *Alpinia zerumbet* JX088668, *Alpinia oxyphylla* NC_035895.1, *Curcuma flaviflora* KR967361, *Curcuma roscoeana* NC_022928.1, *Curcuma longa* MK262732, *Kaempferia galanga* MK209001, *Kaempferia elegans* MK209002, *Zingiber spectabile* JX088661, *Amomum kravanh* NC_036935.1, *Amomum compactum* NC_036992.1, *Amomum villosum* MK262730, *Stahlianthus involucratus* MK262725, *Hedychium coronarium* MK262736, *Costus pulverulentus* KF601573, *Costus viridis* MK262733, and *Canna indica* KF601570.

## References

[CIT0001] Kumar S, Stecher G, Tamura K 2016 MEGA7: molecular evolutionary genetics analysis version 7.0 for bigger datasets. Mol Biol Evol. 33:1870–1874. 27004904 10.1093/molbev/msw054 PMC8210823

[CIT0002] Li X, Hu Z, Lin X, Li Q, Gao H, Luo G, Chen S 2012 High-throughput pyrosequencing of the complete chloroplast genome of *Magnolia officinalis* and its application in species identification. Yao Xue Xue Bao. 47:124–130. 22493817

[CIT0003] Li DM, Zhao CY, Liu XF 2019a Complete chloroplast genome sequences of *Kaempferia galanga* and *Kaempferia elegans*: molecular structures and comparative analysis. Molecules. 24:474. 10.3390/molecules24030474 PMC6385120 30699955

[CIT0004] Li DM, Wu W, Liu XF, Zhao CY 2019b Characterization and phylogenetic analysis of the complete chloroplast genome sequence of *Costus viridis* (Costaceae). Mitochondrial DNA Part B. 4:1118–1120. 10.1080/23802359.2019.1664343 PMC7706813 33365816

[CIT0005] Thompson JD, Gibson TJ, Plewniak F, Jeanmougin F, Higgins DG 1997 The CLUSTAL_X windows interface: Flexible strategies for multiple sequence alignment aided by quality analysis tools. Nucleic Acids Res. 25:4876–4882. 9396791 10.1093/nar/25.24.4876 PMC147148

[CIT0006] Wu D, Larsen K 2000 Zingiberaceae vol 24. Flora of China. Beijing: Science Press p. 322–377.

[CIT0007] Wu D, Liu N, Ye Y 2016 The Zingiberaceous resources in China. Wuhan: Huazhong University of Science and Technology University Press p. 89.

